# Speech Reception Threshold Estimation via EEG‐Based Continuous Speech Envelope Reconstruction

**DOI:** 10.1111/ejn.70083

**Published:** 2025-03-27

**Authors:** Heidi B. Borges, Johannes Zaar, Emina Alickovic, Christian B. Christensen, Preben Kidmose

**Affiliations:** ^1^ Eriksholm Research Centre Snekkersten Denmark; ^2^ Department of Electrical and Computer Engineering Aarhus University Aarhus Denmark; ^3^ Hearing Systems, Department of Health Technology Technical University of Denmark Kongens Lyngby Denmark; ^4^ Department of Electrical Engineering Linköping University Linköping Sweden

**Keywords:** EEG measures, envelope tracking, neural decoding, speech in noise, speech intelligibility

## Abstract

This study investigates the potential of speech reception threshold (SRT) estimation through electroencephalography (EEG) based envelope reconstruction techniques with continuous speech. Additionally, we investigate the influence of the stimuli's signal‐to‐noise ratio (SNR) on the temporal response function (TRF). Twenty young normal‐hearing participants listened to audiobook excerpts with varying background noise levels while EEG was recorded. A linear decoder was trained to reconstruct the speech envelope from the EEG data. The reconstruction accuracy was calculated as the Pearson's correlation between the reconstructed and actual speech envelopes. An EEG SRT estimate (SRT_neuro_) was obtained as the midpoint of a sigmoid function fitted to the reconstruction accuracy versus SNR data points. Additionally, the TRF was estimated at each SNR level, followed by a statistical analysis to reveal significant effects of SNR levels on the latencies and amplitudes of the most prominent components. The SRT_neuro_ was within 3 dB of the behavioral SRT for all participants. The TRF analysis showed a significant latency decrease for N1 and P2 and a significant amplitude magnitude increase for N1 and P2 with increasing SNR. The results suggest that both envelope reconstruction accuracy and the TRF components are influenced by changes in SNR, indicating they may be linked to the same underlying neural process.

## Graphical abstract

This study estimated the speech reception threshold from EEG (SRT_neuro_) using envelope reconstruction of continuous‐speech stimuli. It also investigated the influence of the signal‐to‐noise ratio (SNR) on the temporal response function (TRF). The SRT_neuro_ was within 3 dB of the behaviorally measured SRT (SRT_beh_) for all 20 participants. The TRF analysis showed significant decreases in P2 and N1 latency and increases in their magnitudes with increasing SNR.

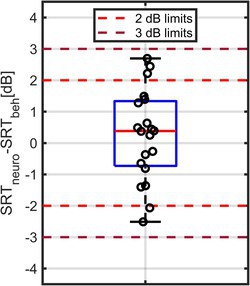

**Abbreviations**
EEG: electroencephalography
HINT: hearing‐in‐noise test
HL: hearing level
ICA: independent component analysis
LR: likelihood ratio
OLSA: Oldenburg Sentence Test
SD: standard deviation
SNR: signal‐to‐noise ratio
SRT: speech reception threshold
SRT_beh_
: behaviorally measured SRT
SRT_neuro_
: SRT estimated from EEG
TRF: temporal response function

## 1 Introduction

Globally, an estimated 430 million people require hearing assistance, with projections suggesting this number will reach 711 million by 2050 (WHO 2021). Hearing loss leads to communication difficulties and can have adverse effects on the individual, including social isolation, depression, and cognitive decline (Li et al. 2014; Lin et al. 2013; WHO 2021). Although hearing aids can alleviate the challenges caused by hearing loss by providing amplification, speech intelligibility in noisy environments typically remains a challenge for hearing‐impaired listeners with hearing aids. Measures of an individual's speech‐in‐noise ability can thus be advantageous for better hearing‐aid fitting, especially with regard to speech‐enhancement processing (Zaar et al. 2024). Speech intelligibility in noise is quantified by the so‐called speech reception threshold (SRT), which is a measure of the signal‐to‐noise ratio (SNR) at which 50% speech intelligibility is achieved. It is typically measured behaviorally, that is, by presenting speech stimuli in background noise and asking the test participant to repeat back what they understood. However, this method has limitations, particularly its reliance on the participant's ability and willingness to respond. Previous studies have shown promise in estimating the SRT using brain responses to matrix sentences recorded with electroencephalography (EEG) (Lesenfants et al. 2019; Vanthornhout et al. 2018). If the SRT can be accurately predicted from EEG, it implies that no active response is necessary. This could facilitate the measurement of SRTs, particularly in populations that cannot respond, such as infants and unconscious patients (Lesenfants et al. 2019; Van Hirtum, Somers, Dieudonné, et al. 2023; Vanthornhout et al. 2018) and assist in evaluating the impact of hearing‐aid algorithms.

Through recent years, it has become possible to study neural responses to continuous stimuli. This is usually achieved with decoding (backward‐modeling) and encoding (forward‐modeling) models (Alickovic et al. 2019). Additionally, a combination of these approaches called canonical component analysis (de Cheveigné et al. 2018; Gillis, Van Canneyt, et al. 2022) or neural networks (Accou et al. 2023; Puffay, Vanthornhout, et al. 2023; Puffay, Accou, et al. 2023) can be used. Both linear decoding and encoding approaches have been used to predict speech intelligibility (Lesenfants et al. 2019; Vanthornhout et al. 2018). Although the decoding model is a strong model, predicting one speech feature from multiple EEG channels (Alickovic et al. 2019), it lacks the ability to investigate the spatiotemporal pattern of the correlation (Haufe et al. 2014). The encoding model, by contrast, predicts the EEG channels from sound features (Alickovic et al. 2019) and thus yields a temporal response function (TRF) for each of the EEG channels. The TRF represents the “impulse response” that connects the sound feature at the input to the EEG channel signal at the output. Because the TRFs are from individual channels, the encoding approach allows for interpretation of the spatiotemporal pattern of brain activity (represented in the EEG signal) evoked by the stimulus feature. The TRF is akin to the event‐related potential, but instead of having to repeat stimuli multiple times, the TRF allows to estimate how the brain responds to continuous stimuli. The TRF furthermore represents a response to specific stimuli feature, whereas the ERP is a representation of the cumulative EEG response (Crosse et al. 2021).

There is a well‐established relationship between acoustic and linguistic features of the stimuli and brain activity related to the processing thereof termed “neural speech tracking” (Luo and Poeppel 2007; Obleser and Kayser 2019; Viswanathan et al. 2019). A simple way of measuring neural speech tracking is through reconstruction of the stimuli from the EEG signal with a decoder (Brodbeck and Simon 2020). The speech envelope is robustly tracked, making it a good potential candidate for a clinical assessment (Vanthornhout et al. 2018). Although there is evidence that envelope tracking is a prerequisite for speech intelligibility (Riecke et al. 2018; Verschueren et al. 2019; Wilsch et al. 2018), it is not a direct measure of speech intelligibility and, therefore, not a sufficient condition for speech intelligibility.

The objective of this study is to investigate the feasibility of EEG‐based SRT estimation. One concern when conducting audiological testing is whether the results are representative of the patient's performance in their everyday sound environment. To address this, employing more naturalistic stimuli will enhance the ecological validity of the test. Therefore, instead of employing matrix sentences for speech testing and EEG measurements, as done in previous studies (Lesenfants et al. 2019; Vanthornhout et al. 2018), we use open‐set sentences for speech testing and excerpts from an audiobook for EEG measurements to enhance the ecological validity of the target speech stimuli. Additionally, we aim to explore how changes in SNR affect the TRF in a young, normal‐hearing population when using continuous speech. Previous research involving 5‐year‐olds has shown changes in the latency and amplitude of the P1 deflection of the TRF in response to changes in SNR of continuous speech in stationary speech‐weighted noise (Van Hirtum, Somers, Verschueren, et al. 2023). As the N1 and P2 components are observed in the auditory evoked potential in the developed auditory system (Ponton et al. 2000), it is worthwhile investigating whether they manifest themselves in the TRF and how they vary across SNRs. A previous study has reported that the latencies of TRF deflections decrease, and their amplitudes increase with higher SNR levels when using recordings from magnetoencephalography (MEG) (Ding and Simon 2013). One study with young normal hearing individuals showed that the TRF amplitudes, measured using EEG, were higher across the entire scalp when speech intelligibility was at 100% compared to lower speech intelligibility levels (Verschueren et al. 2020). The study also revealed that the latency of the TRF peaks decreased as speech intelligibility increased when using matrix sentences as stimuli. Building upon these studies, we hypothesize that changes in the SNR when using continuous speech as stimuli will induce changes in the TRF in young, normal‐hearing listeners as well.

## 2 Material and Methods

### 2.1 Participants

Inclusion of participants was based on the following inclusion criteria: native Danish speakers, right‐handed, no neurological diseases, and no dyslexia to a degree that significantly affects their everyday lives, aged between 18 and 30 years, and normal hearing. Inclusion was based on self‐reporting, except for the normal hearing criterion, which was confirmed based on pure‐tone audiometry. Normal hearing was defined as hearing thresholds of maximally 20 dB hearing level (HL) across frequencies ranging from 0.125 to 8 kHz (0.125, 0.25, 0.5, 0.75, 1, 1.5, 2, 3, 4, 6, and 8 kHz), consistent with WHO guidelines for normal hearing (WHO 2021). The hearing level was assessed in 5‐dB steps. The participants received compensation in the form of gift cards and transport reimbursement for their participation. Prior to participation, all participants provided signed and informed consent. The study was approved by the Institutional Review Board at Aarhus University under approval number TECH‐2022‐004.

### 2.2 Experiments

The experiment was divided into three visits. During the first visit, the participants' SRT was measured behaviorally (SRT_beh_), a reading span test (Daneman and Carpenter 1980) and Edinburgh Handedness Inventory test (Oldfield 1971) were conducted, and an ear impression was made. The data recorded during the second visit are intended for another study and are therefore not further described here. During the third visit, the EEG experiment was conducted.

#### 2.2.1 EEG Recordings

EEG data were recorded at a sampling rate of 4096 Hz using a BioSemi Active EEG (Amsterdam, Netherlands) setup with a 64‐electrode EEG‐cap and in addition two mastoid electrodes. Additionally, 12 ear‐EEG electrodes and one Fpz electrode were recorded with the SAGA32+/64 + system (TMSi, Oldenzaal, Netherlands). However, analysis of the ear‐EEG data is not included in this study and has been deferred for future investigation. This article only presents results from the analysis of scalp electrodes.

#### 2.2.2 Stimuli Presentation

In the EEG paradigm, the diotic stimuli were presented via a soundcard (RME Hammerfall DSP multiface II, Audio AG, Germany) and delivered through Etymotic ER‐1 insert Earphones (Etymotic Research Inc., IL, USA) connected to the ear‐EEG earpieces via sound tubes. The experiment was conducted in a double‐shelled RF shielded test box. The system was calibrated using an ear simulator type 4157 and the outer‐ear simulator DB2012 (Brüel & Kjær, Denmark), with measuring amplifier type 2610/2636 (Brüel & Kjær, Denmark). During stimuli presentation, the speech was maintained at 65 dB SPL, but the noise level was varied to obtain the desired SNRs. The steady‐state speech‐shaped noise used as noise in both the behavioral SRT and EEG experiment was used for calibration. The stimuli were presented in the same way in both the EEG experiment and the behavioral speech test, with the only difference that the stimuli were presented through disposable foam ear tips in the speech test and through the ear‐EEG earpieces during the EEG recordings.

#### 2.2.3 Speech Test

SRT_beh_ was assessed using the Danish hearing‐in‐noise test (HINT) sentence lists (Nielsen and Dau 2011), each consisting of 20 five‐word meaningful sentences. The SNR at which 50% of the words were correctly repeated was chosen as the SRT_beh_ and determined using an adaptive procedure. In the adaptive procedure, the SNR for the next sentence was updated by adjusting the noise level by ∆L (in dB) relative to the noise level for the previous sentence. ∆L was calculated according to $∆L=\left(p_{\text{previous}}-p_{\text{target}}\right)*g$, where $p_{\text{previous}}$ is the proportion of words correct repeated in response to the previous sentence, $p_{\text{target}}$ is the target proportion (here 0.5), and *g* is a constant, which was set at 8 dB for the first 4 sentences and at 4 dB for the remaining sentences (Bio‐logic Systems Corp. 2005). The resulting changes in SNR depending on words correct repeated are shown in Table 1. This approach for adaptive SNR changes when using word scoring is similar to approaches described by Hagerman and Kinnefors (1995), Hernvig and Olsen (2005), and Brand and Kollmeier (2002). Two concatenated HINT sentence lists (i.e., 40 sentences) were used in the adaptive procedure, with the first sentence starting at a SNR of −10 dB. The SRT_beh_ was calculated as the mean SNR obtained with sentences 5–40, that is, responses obtained with the smaller constant of g (4 dB).

**TABLE 1:** SNR changes depending on words correct.

Words correct	SNR change [dB] for sentence 1–4	SNR change [dB] for sentence 5–40
0	4	2
1	2.4	1.2
2	0.8	0.4
3	−0.8	−0.4
4	−2.4	−1.2
5	−4	−2

*Note:* SNR changes depending on the number of words correctly answered. Note that the step size is halved after the first 4 sentences.

Initially, two concatenated HINT training sentence lists were introduced to the participant using an adaptive procedure. This was followed by two concatenated HINT sentence lists to adaptively determine the SRT_beh_. After the SRT_beh_ was determined, one HINT sentence list was run for each of 5 different SNR levels: SRT_beh_ + 4, SRT_beh_ + 2, SRT_beh_, SRT_beh_ − 2 and SRT_beh_ − 4 dB, as well as for a clean‐speech condition, to obtain the words‐correct score data. In the following, only the adaptively measured SRT_beh_ was used, whereas the word‐scoring data measured in the six mentioned conditions were not further processed.

#### 2.2.4 EEG‐Based SRT Estimation

**FIGURE 1:**
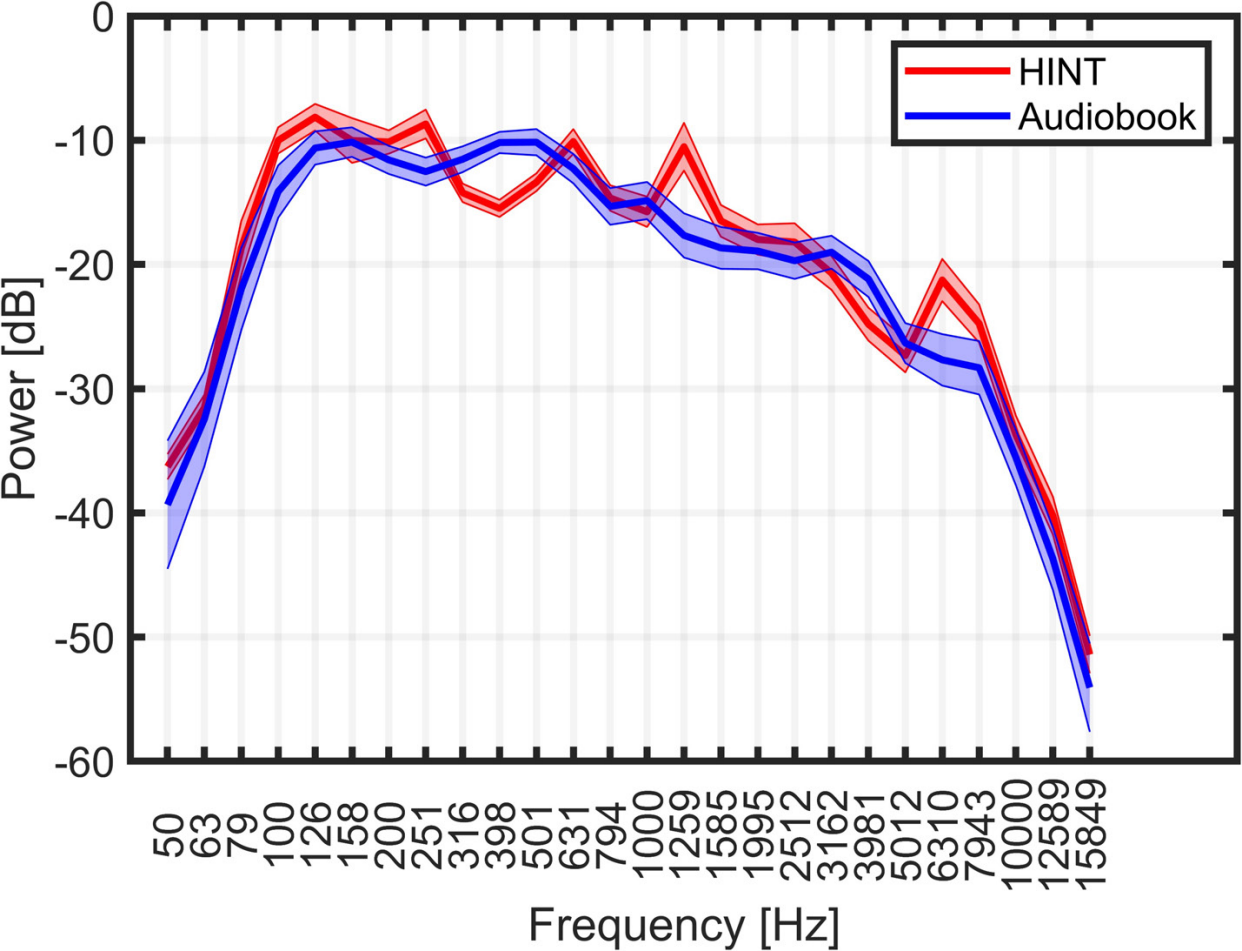
The one‐third octave band long‐term spectral density for the HINT sentences and the audiobook material. The bold lines represent the mean spectral density, the shaded areas around them depict the range of ±2 standard deviations (SD) across the audio excerpts (audiobook material) or lists (HINT sentences).

To estimate the SRT_beh_ from EEG (SRT_neuro_), audiobook excerpts were used as stimuli. Audiobooks provide naturally connected speech, offering better ecological validity than alternative options like concatenated HINT sentences. The audiobook excerpts of approximately 1‐min duration, taking sentence boundaries into account, were presented to the participants at 5 different SNR levels relative to the individual participants' SRT_beh_ (SRT_beh_ + 4, SRT_beh_ + 2, SRT_beh_, SRT_beh_ − 2, SRT_beh_ − 4 dB) and clean speech. In analogy to the behavioral speech test stimuli, the SNRs were obtained by using a fixed speech level of 65 dB SPL and adjusting the noise level. The audiobook material was filtered using a first‐order low‐pass filter with a cut‐off frequency of 2 kHz to approximate the power spectral density of the HINT material. Figure 1 illustrates the resulting mean third‐octave band power spectral density for the audiobook, along with that of the HINT speech corpus. The power spectral density was extracted from each audio excerpt (~1 min duration per excerpt) and from each HINT list by concatenating all HINT sentences belonging to the list (~30 s duration per list).

The average fundamental frequency was 123 Hz for the audiobook material and 117 Hz for the HINT material. The noise started 2000 ms prior to the onset of the audiobook sound and ended 600 ms after the offset of the audiobook sound. The noise was faded in and out with a 400‐ms raised‐cosine ramp‐on and ramp‐off. The experiment consisted of two training trials to familiarize the participant with the task. Hereafter, 16 trials, each approximately 1 min long, were recorded for each condition, resulting in a total of 96 trials. The amount of data collected per condition was identical. SNR conditions and clean speech were randomly presented within each block. To keep the participants motivated and attentive to the auditory stimuli, a content‐related 2‐AFC (two‐alternative forced‐choice) yes/no question was asked after each trial. A recreational activity was included between the blocks to help keep the participants engaged and attentive. This activity involved a small, unrelated task, such as describing one's morning routine in detail.

### 2.3 Data Analysis

#### 2.3.1 Stimuli Preprocessing

All analysis was conducted using Matlab (MathWorks Inc.) and the mTRF Toolbox (Crosse et al. 2016). The audiobook stimuli were processed by extracting the broadband amplitude envelope as the absolute value of the analytical signal obtained via the Hilbert transform. A power law was applied according to (𝑡)^0.6^, where *x* is the envelope and *t* is time, to compensate for the compression in the auditory system (Biesmans et al. 2017). The signal was then bandpass filtered between 1 and 8 Hz using third‐order high‐ and low‐pass Butterworth filters, which were employed forward and backward using Matlab's “filtfilt” function, therefore overall resulting in a 12th‐order bandpass filter, and finally down‐sampled to a sampling rate of 64 Hz.

#### 2.3.2 EEG Preprocessing

The EEG data were processed in two separate ways: one to optimize the independent component analysis (ICA) weights (the “ICA pipeline”) and another to process the EEG data for the decoder (the “EEG pipeline”). To obtain the ICA weights, the pipeline involved down‐sampling the data to 256 Hz to reduce processing time. This was followed by bandpass filtering between 1 and 100 Hz using third‐order high‐ and low‐pass Butterworth filters, again employed forward and backward using Matlab's “filtfilt” function, resulting in a 12th‐order bandpass filter. Subsequently, a Zapline notch filter was applied, as described by de Cheveigné (2020). Channels with an RMS value larger than 3 times the mean RMS value across all channels were labeled as bad channels and removed. The removed channels were replaced using spherical interpolation. The channels were re‐referenced to their common average, and all trials were epoched and concatenated. Finally, the “runica” function from eeglab (Delorme and Makeig 2004) was applied to obtain ICA weights.

The EEG pipeline was applied by first down‐sampling the EEG data to 256 Hz, removing the channels labeled as bad in the ICA pipeline, and reconstructing them using spherical interpolation. The EEG signal was then re‐referenced to the average of all channels. Next, the ICA weights were applied to the data, and any components labeled as ≥ 75% likely to be artifacts from eyes, heart, line noise, or channel noise by the IClabel algorithm (Pion‐Tonachini et al. 2019) were removed. Following this, the data were bandpass‐filtered between 1 and 8 Hz using third‐order high‐ and low‐pass Butterworth filters, which were employed forward and backward using Matlab's filtfilt function. Then the data were resampled to 64 Hz and normalized by dividing each channel with the mean of the standard deviation (SD) across all channels. Finally, the channels were referenced to the average of all channels.

#### 2.3.3 SRT Estimation

A decoder was used to estimate the SRT_neuro_, with training and testing conducted at the participant level. The decoder was trained using all 16 trials from the clean speech condition (no noise). The decoder was then applied to the trials from the noise conditions, where the envelope was reconstructed for each individual trial. The reconstruction accuracy was calculated as the Pearson's correlation between the reconstructed envelope and the actual envelope of the speech stimulus. To train the decoder, a decision window of $\tau =\left[-100\;350\right]$ ms was used and the regularization parameter, λ, were optimized in the range $\left[10^{-4}-10^{10}\right]$. One λ value per participant was used for decoder training. The participant's specific λ value was determined by selecting the value yielding the greatest reconstruction accuracy when performing a leave‐one‐out cross validation of all the trials in the clean speech condition. A one‐sided permutation test was performed with a precision of 0.01 and α = 0.05 to determine whether the reconstruction accuracy increased with increasing SNR at the population level (in general for all participants). To that end, the following conditions were tested against each other: SRT_beh_ − 4 dB and SRT_beh_ − 2 dB; SRT_beh_ − 2 dB and SRT_beh_; SRT_beh_ and SRT_beh_ + 2 dB; SRT_beh_ + 2 dB and SRT_beh_ + 4 dB.

The noise floor was determined by using mismatched envelopes from 68 audio excerpts. These excerpts were extracted from the same audiobook as the stimuli presented to the participants, underwent the same preprocessing, and were of approximately the same length. However, they were not part of the speech stimuli presented to the participants. Reconstruction accuracy was then calculated as the Pearson's correlation between the reconstructed envelope from each trial with noise (80 trials) and the 68 mismatched envelopes. To ensure equal length of the two envelopes, the EEG used for envelope prediction, or the mismatched envelope was cut to the length of the shortest duration of the two. Subsequently, the mean reconstruction accuracy was calculated across trials to obtain one noise floor value per participant. To find a reconstruction accuracy for the clean speech condition, leave‐one‐out training was used on trials only from that condition.

**FIGURE 2:**
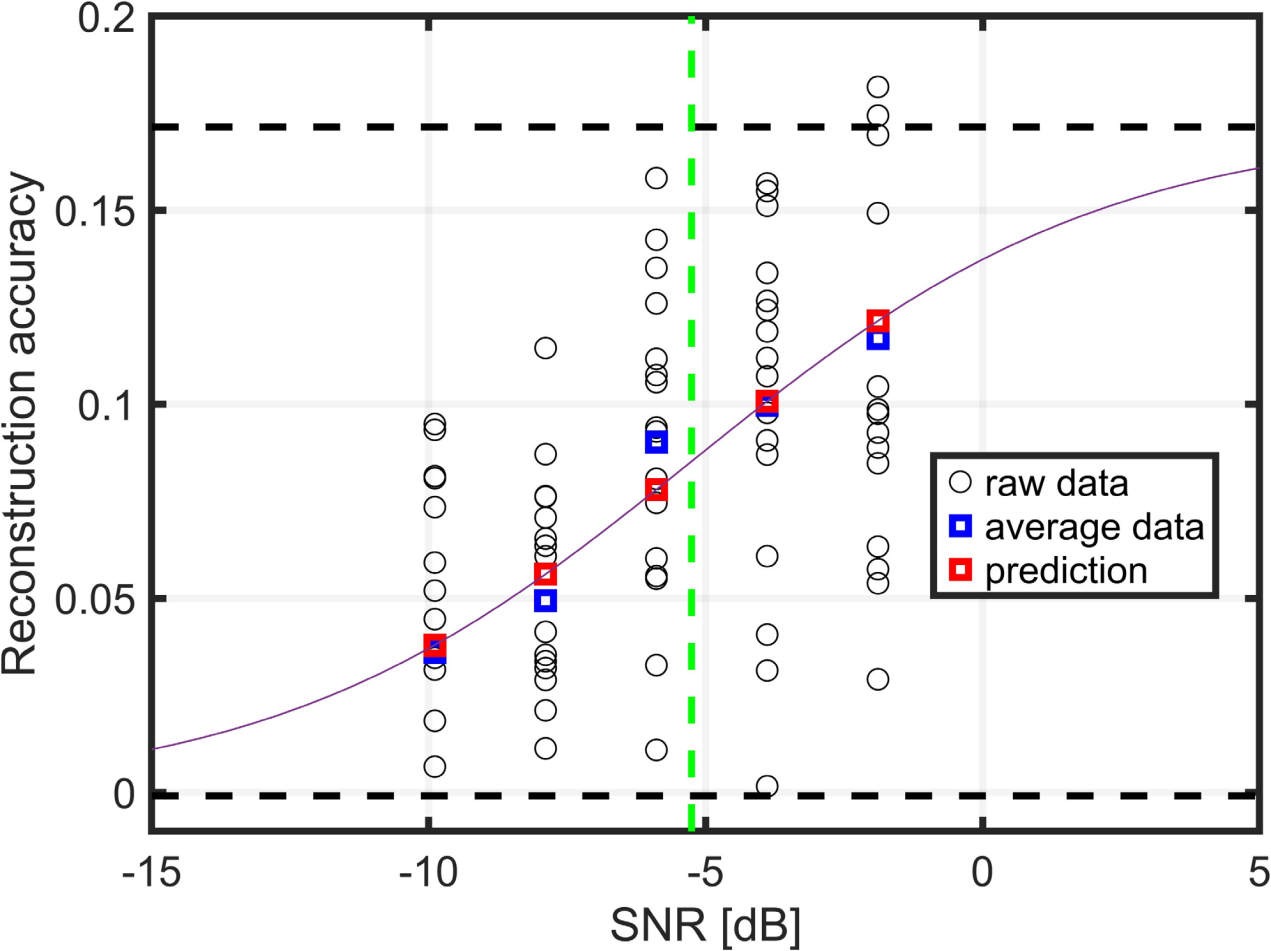
Example of a sigmoid fit for the reconstruction accuracy‐versus‐SNR data for one participant. Each circle represents the reconstruction accuracy for a single trial, the blue squares represent the average reconstruction accuracy for each SNR condition, and the red squares show the datapoints predicted by the function for the considered SNR conditions. The dashed black lines represent the p and b coefficients of the sigmoid fit and the green dashed line the m coefficient of the sigmoid fit used for the SRT_neuro_ determination.

A sigmoid function described in (Farris‐Trimble and McMurray 2013) was fitted to the reconstruction accuracy‐versus‐SNR data from single participants. The sigmoid function is parameterized as

(1) $$S\left(\mathrm{SNR}\right)=\frac{p-b}{1+\exp\left(4\cdot \frac{s}{p-b}\cdot \left(m-\mathrm{SNR}\right)\right)}+b$$

where *p* is the mean reconstruction accuracy of the clean speech condition, *b* is the mean of the reconstruction‐accuracy noise floor, *s* is the slope and *m* is the midpoint value, the midpoint value is used as the SRT_neuro_. We hypothesize that this is a potentially biased estimator of SRT_beh_, because reconstruction accuracy and % of words correct are not the same parameter. Thus, implementation of an offset from the midpoint of the fitted sigmoid function might be necessary to estimate SRT_beh_. During the fitting procedure, boundaries of [$-10^{10}$, $10^{10}$] were imposed for the m value and [$0,10^{10}$] for the *s* value. To ensure that the fit was conducted on an increasing trend, fits were only performed for participants for whom the reconstruction accuracies of the clean speech condition were significantly higher than the noise floor. Here, the mean reconstruction accuracy of the noise floor within trials was used to test against the clean speech reconstruction accuracies such that 16 datapoints were tested against 16 datapoints. This was tested using a permutation test with 5% significance level; all participants fulfilled this criterion. Each fitting was then repeated 100 times with a random initialization point for the *m* value ±10dB from −2.52 dB, the average SRT_beh_ value measured for normal‐hearing listeners found in the validation of the Danish HINT test (Nielsen and Dau 2011). The *m* parameters initialization point was randomized to avoid biasing it toward a specific point while still initializing the fit within a reasonable distance to a realistic value. The slope values were always initialized at 0. This SRT_beh_ was based on sentence‐based scoring, because no word‐based scoring was reported. Subsequently, the average of the sigmoid parameters for the 10 fits with the highest $R^{2}$ values was chosen as the final fit. This procedure was used to mitigate issues related to local minima and enhance robustness of the fitting method. An example of a fit using data from a representative participant is shown in Figure 2. The entire described fitting procedure was repeated 100 times to assess the robustness of the fitting method.

#### 2.3.4 TRF

To compute the TRF, an encoder was trained using leave‐one‐out training within each condition. A hyperparameter (λ) value of 100 was used for all participants, as it yielded the highest mean prediction accuracy across participants and SNRs when testing the hyperparameters in the range $\left[10^{-6}-10^{5}\right]$. The λ was fixed not only across conditions but also across participants to facilitate the comparison of TRFs across conditions and participants. The time window investigated, $\tau =\left[-100\;500\right]\;\mathrm{ms}$, was chosen to include the major components of the TRF. Model performance was assessed by calculating Pearson's correlation between the predicted and the true EEG signal, termed prediction accuracy. A global selection of 17 electrodes was chosen based on high prediction accuracies, mirroring an approach used in a previous study (Fuglsang et al. 2017). A permutation test was performed to assess whether the mean prediction accuracy of the 17 electrodes in each condition, on a participant level, significantly exceeded that of the noise floor (α = 0.05). The noise floor was calculated using the encoder model and mismatched envelopes, akin to the approach used in the decoding analysis. Only the TRFs with significant prediction accuracies were included in the further analysis. The mean TRF was calculated from the 17 chosen electrodes for each participant and SNR. Then the latencies and amplitudes of the two positive deflections (P1 and P2) and the one negative deflection (N1) were found by fitting a Gaussian function: $a\left(t\right)=p*\exp\left(-{\left(\frac{t-l}{w}\right)}^{2}\right)$, where *a* is amplitude, *p* is the deflection, *t* is time, *l* is the latency of the deflection, and *w* is the width of the deflection. The boundary parameters used for the fit are shown in Table 2.

**TABLE 2:** Boundary parameters.

Deflection	*p* [a.u.]	l [ms]	w [ms]
P1	[0 5]	[0 100]	[0 50]
N1	[−5 0]	[70 150]	[0 50]
P2	[0 5]	[100 300]	[0 50]

*Note:* Boundary parameters for Gaussian function fit on the TRFs.

The Gaussian function fit was excluded from further analysis if it obtained an $R^{2}$ value of less than 0.5. The relationship between the SNR, latency, and amplitude for the different deflections was assessed using a linear mixed model in RStudio (Posit team, Boston, MA, Version 2023.12.1.402) with the nlme package (Lindstrom and Bates 1990; Pinheiro and Bates 1996) and maximum likelihood criteria. Participant number was modeled as a random effect (P), allowing for an individual offset for each participant. The latency and amplitude features (F) of the deflections were estimated using the model: F ~ SNR + (P|1). Residuals were analyzed for normality by observing the qq‐plot and histogram of residuals. Statistical significance of the fixed effect was assessed with a univariate Wald test with α = 0.05. A Holm–Bonferroni correction was applied to avoid familywise errors, as six significance tests were performed, one for each feature. The clean speech condition was not included in the statistical test. A grand average TRF was calculated for illustration purposes by averaging the TRFs across subjects in each condition.

## 3 Results

### 3.1 SRT Estimation

**FIGURE 3:**
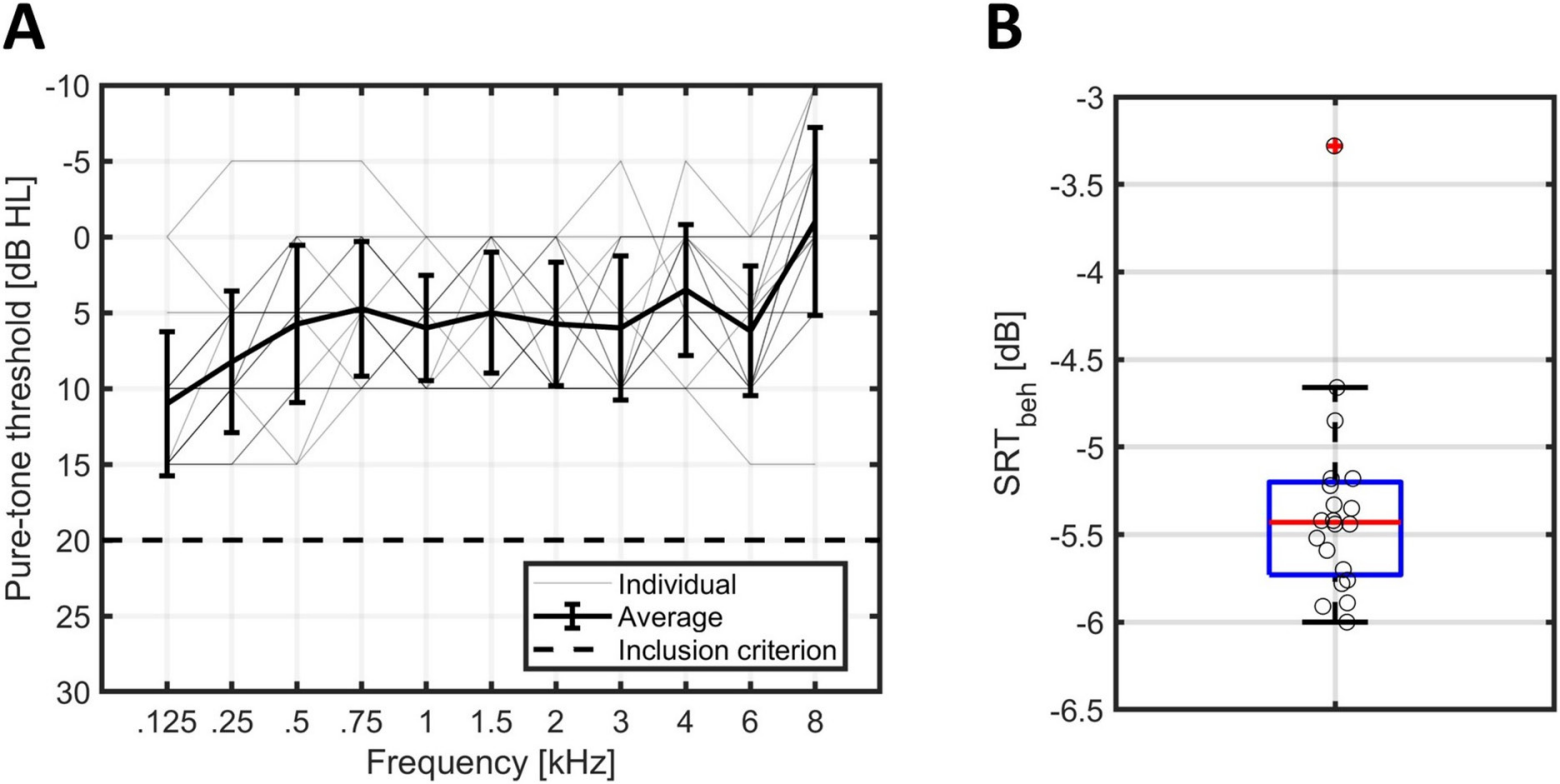
(A) Highest pure‐tone threshold across ears for each of the participants (thin lines), along with the mean and standard deviation across participants (shown in bold lines). The inclusion criteria of max 20 dB hearing level is shown as a dashed line. (B) Boxplot of the measured SRT_beh_. The red line shows the median, the edges of the blue box the 25th and 75th percentiles, the black whiskers mark the most extreme values for non‐outliers, and the red cross marks datapoints considered outliers, defined as a value more than 1.5 times the interquartile range away from the bottom or top of the box. Data for individual participants are shown as circles and are jittered slightly along the x‐axis to improve the readability of the figure.

Twenty‐two normal hearing participants were recruited for this study. However, due to technical problems, data recordings from two participants were incomplete, leading to their exclusion from the analysis. Consequently, the final analysis included data from 20 participants, comprising 17 females and 3 males, aged 18–29 years (mean 24.3 years). The highest pure‐tone threshold across the two ears is shown as a function of audiometric frequency in Figure 3A. The $\mathrm{SR}T_{\mathrm{beh}}$ ranged from −6.00 to −3.28 dB with a mean of −5.35 dB and are shown in Figure 3B.

**FIGURE 4:**
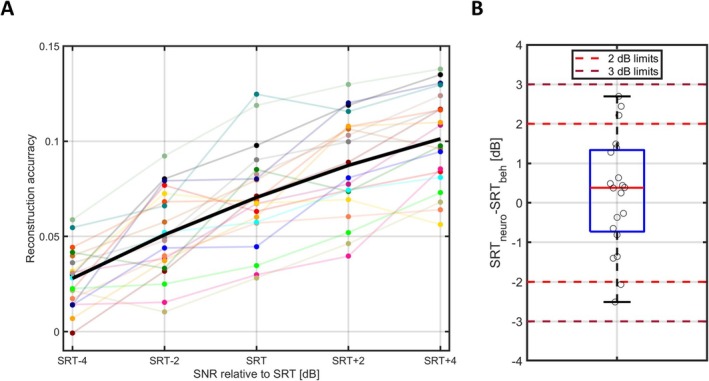
(A) Reconstruction accuracy as a function of SNR (relative to the individual participant's specific SRT_beh_) for each individual (dots, connected by thin lines) and the mean across participants (bold line). (B) Boxplot of the difference between the SRT_neuro_ and SRT_beh_. The solid red line shows the median, the edges of the blue box the 25th and 75th percentiles, and the black whiskers mark the most extreme values. Data for individual participants are shown as black circles and are jittered along the x‐axis. The light red and dark red dashed lines indicate an absolute difference of 2 and 3 dB, respectively.

To investigate the feasibility of estimating the SRT from EEG measurements, we analyzed the reconstruction accuracy as a function of the SNR. Figure 4A shows the reconstruction accuracy as a function of SNR (relative to the behaviorally measured SRT) for each participant (thin lines) as well as the mean across participants (bold line). The mean reconstruction accuracy exhibits a monotonic increase. Permutation tests showed a significant increase in the reconstruction accuracy for each 2 dB increase in SNR (all *p* values below $10^{-3}$).

The individual SRT_beh_ (shown in Figure 3B) was used to determine the SNR for the audiobook stimuli employed in the EEG recordings. With the SRT_beh_ ranging from −6.00 to −3.28 dB, the corresponding SNRs for the individual participants spanned from −6.00 ± 4 dB to −3.28 ± 4 dB in 2 dB steps (SNRs of −10–0.72 dB). The difference between the SRT_neuro_ and the SRT_beh_ is shown in Figure 4B. The median of the individual difference between SRT_neuro_ and SRT_beh_ was very close to zero (0.38 dB), with group‐level median values of −5.43 dB for SRT_beh_ and −5.17 dB for SRT_neuro_. The standard deviation of the difference was 1.45 dB. We found that 15 out of the 20 participants had a difference between SRT_neuro_ and SRT_beh_ within ±2 dB and all participants had a difference between SRT_neuro_ and SRT_beh_ within ±3 dB. The maximum difference of the fitting procedure when repeating it 100 times within participants was 1.42 dB.

### 3.2 TRF

**FIGURE 5:**
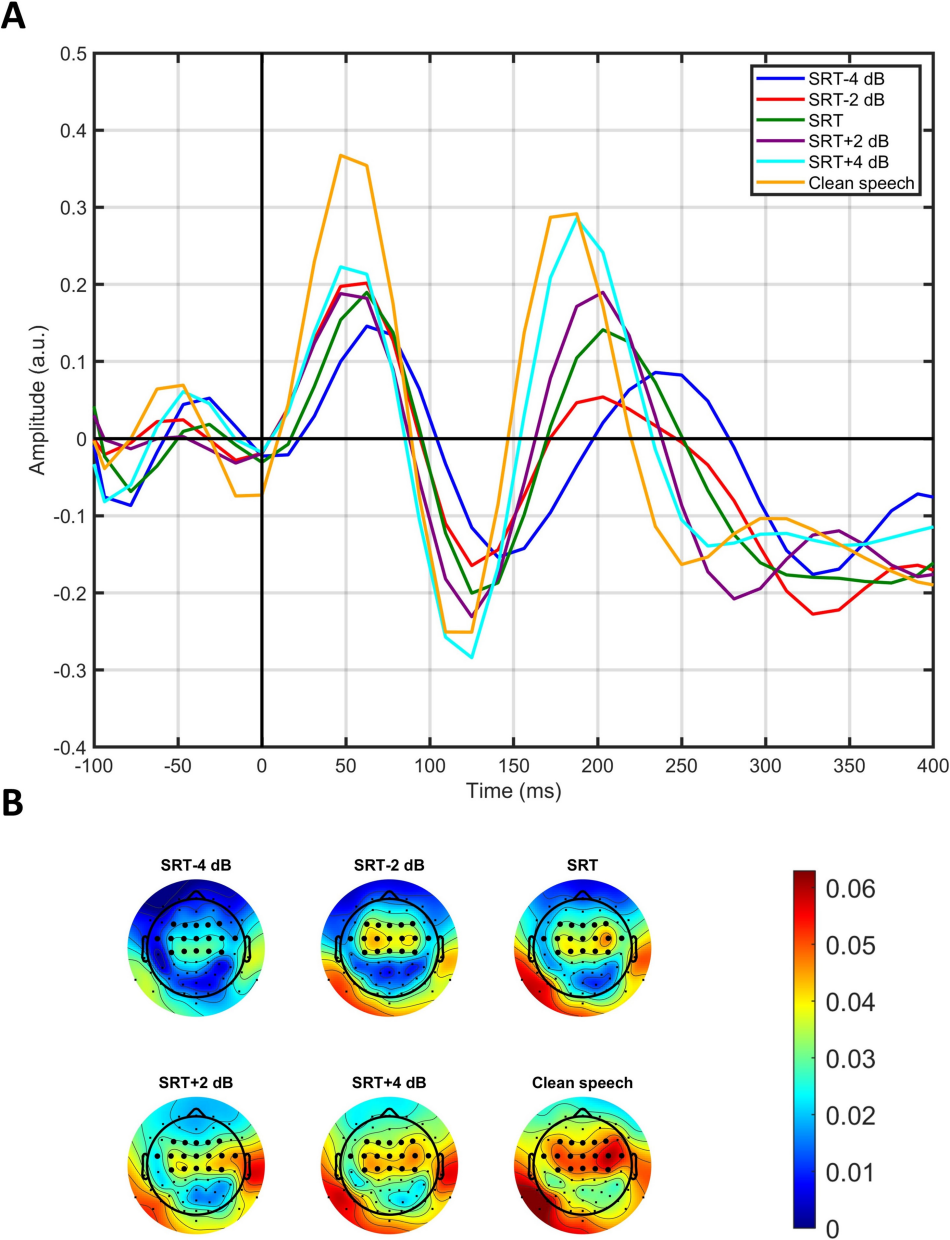
(A) The grand average TRF in a −100–400 ms time window for each of the six SNR conditions; the last 100 ms of the time window is not depicted due to edging effects when approaching 500 ms. (B) The grand average prediction accuracy from the encoding model; the electrodes used for the TRF analysis are shown in bold.

**FIGURE 6:**
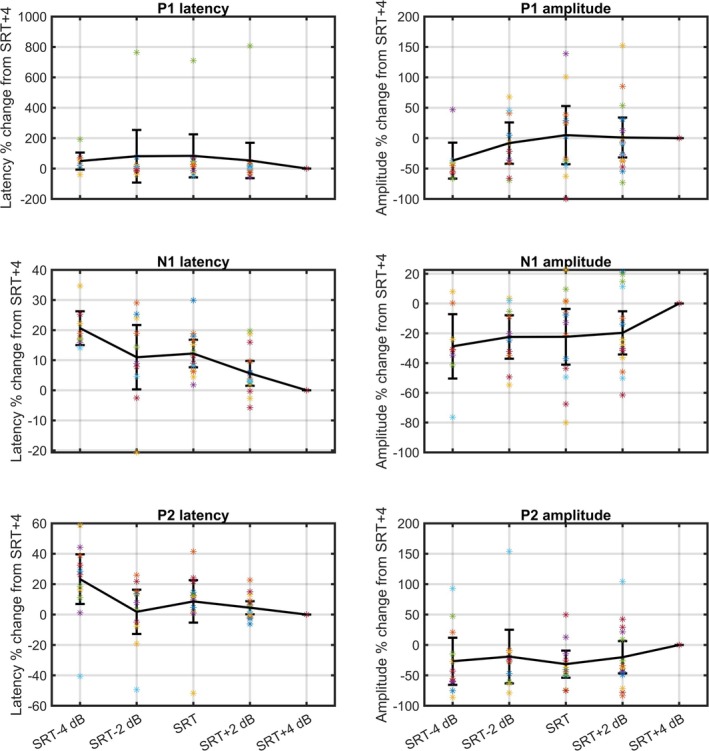
Percentage of change in latency and amplitude for the SNR conditions compared to the highest SNR condition (SRT + 4 dB). The across‐participant average of the percentage of change in latency and amplitude of the TRF deflections is plotted along with the 95% confidence interval, and the values for individual participants are depicted as colored stars.

The grand average TRF exhibited three distinct deflections within the time window of 0–300 ms, consisting of two peaks around 50 and 200 ms (P1 and P2) and a trough around 120 ms (N1). The grand average TRF is shown in Figure 5A for each of the 5 SNR conditions and the clean speech condition. TRFs for individual participants are provided in Appendix S1. The prediction accuracy values from the encoding model were higher centrofrontally compared to the rest of the scalp, increasing with increasing SNR (Figure 5B). The *p* values obtained from the Wald test and the mixed‐model values for the fixed effects are presented in Table 3. Statistically significant values were observed for the latency and amplitude of N1 and P2. The negative fixed effect coefficient of the P2 (−6.001) and N1 (−1.517) latency indicates that the latencies decreased with increasing SNR. The coefficients of the N1 (−0.015) and P2 (0.018) amplitudes indicate that the magnitude of the components amplitude increased with higher SNR. Figure 6 shows a general pattern of decreasing latencies and enhanced amplitude with increasing SNR.

**TABLE 3:** Statistical results from a linear mixed‐effects model examining latencies and amplitudes.

Component	Parameter	Coefficient	*t*	Degrees of freedom	Std. error	*p*
P1	Amplitude	0.011	2.163	36	0.005	0.037
	Latency	−0.940	−1.201	36	0.782	0.238
P2	**Amplitude**	**0.018**	**2.995**	**36**	**0.006**	**0.005**
	**Latency**	**−6.001**	**−5.204**	**36**	**1.153**	***p* < 10** ^**−3**^
N1	**Amplitude**	**−0.015**	**−3.343**	**36**	**0.004**	**0.002**
	**Latency**	**−1.517**	**−2.533**	**36**	**0.599**	**0.016**

*Note:* Model coefficients, the t‐values, degrees of freedom, standard errors and *p* values for the amplitudes and latencies. Significant results after Holm–Bonferroni correction are marked with bold text.

## 4 Discussion

### 4.1 SRT Estimation

The measured behavioral SRT (SRT_beh_) and the SRT estimated from the EEG data based on the reconstruction accuracy (SRT_neuro_) were almost identical on the group level, with median values of −5.43 dB and −5.17 dB, respectively. When analyzing the results on the level of individual participants, we found that the SRT_neuro_ was within 2 dB from the SRT_beh_ for 15 out of 20 participants (75%) and within 3 dB from the SRT_beh_ for all participants (100%), as shown in Figure 4B. The maximum difference of the fitting procedure when repeating it 100 times within participant was 1.42 dB, indicating the robustness of the fitting procedure. The standard deviation of the difference between SRT_neuro_ and SRT_beh_ was 1.45 dB. It should be noted for reference that the average within‐participant standard deviation for the Danish HINT is 0.86 dB for sentence‐based scoring, resulting in the variation of the difference between test and retest of $0.86*\sqrt{2}=1.22\;\mathrm{dB}$ (Nielsen and Dau 2011; Rønne et al. 2017). The standard deviation of the difference between SRT_neuro_ and SRT_beh_ is 1.45 dB and thus close to the test–retest variation expected for multiple measurements of the SRT_beh_ itself. It is relevant to note that both the SRT_beh_ and SRT_neuro_ estimates are associated with some variance. Specifically, both measures are influenced to some extent by attentional factors (Ding and Simon 2012; Koelewijn et al. 2017; Mesgarani and Chang 2012; O'Sullivan et al. 2015). As the SRT estimates are derived from different experimental paradigms and are therefore not obtained concurrently, variations in attention across paradigms may contribute to the overall variance. As a result, the variance introduced by attentional differences is expected to accumulate, with both SRT_beh_ and SRT_neuro_ contributing to the observed variability. The quality of the EEG recordings can vary as well (Wilroth et al. 2023), contributing to the overall variation between the two measures.

Prediction of SRT from EEG has been done in a study by Lesenfants et al. (2019), in which the SRT_neuro_ was estimated within 2 dB from the SRT_beh_ for 8 out of 19 participants (42%). However, several differences exist between the two studies. The speech material to determine SRT_neuro_, with Lesenfants et al. (2019) using concatenated Flemish matrix sentences instead of the continuous‐speech stimuli. In the current study, the SRT_beh_ values ranged from −6.00 to −3.28 dB (a 2.72‐dB range) with a mean of −5.35 dB, whereas Lesenfants et al. (2019) observed a range of −10.3 to −4.7 (5.6‐dB range) with a mean of −7.1 dB. The narrower range and higher mean SRT_beh_ in the current study can likely be attributed to the use of an open‐set sentence test, whereas Lesenfants et al. used a matrix speech test for determining the SRT_beh_. To predict the envelope of the stimuli, Lesenfants et al. (2019) used a different model, an encoder. Moreover, Lesenfants et al. (2019) used a grand‐average model to reduce experimental time, and each participant had 2 min less EEG data collected with clean speech for model training and 8–10 min less test data per noise condition than in the current study.

The SNRs used in the noise conditions ranged from −12.5 to 2.5 dB in the Lesenfants et al. (2019) study, whereas the SNRs in the current study ranged from −10.00 to 0.72. Thus, the SNR levels are comparable across the two studies, despite the differences in SRTs. In the Lesenfants et al. (2019) study, the SNRs were constant across participants, in contrast to the current study, where the SNRs were chosen in an ordered manner in 2‐dB steps around the SRT_beh_. To assess whether this selection of SNRs potentially biased the midpoint of the sigmoid fits toward the SRT_beh_ in the current study, we applied the methods for estimating the SRT_neuro_ on simulated data with the SNR sampling grid moved from −4 to +4 dB in 1‐dB intervals. Data were simulated 100 times for each of the 9 sets of SNRs. The difference between the estimated SRT_neuro_ and the measured SRT_beh_ was found. Ultimately a one‐way ANOVA test for equal means between the simulated sets of SNR was conducted, and a *p* value of 0.20 was obtained, suggesting no statistical evidence that the SNR selection used biased the sigmoid fit. For further information regarding the simulated data experiment, see Appendix S2.

By centering the SNRs around the SRT_beh_, we increased the probability of measuring meaningful reconstruction accuracy values between flooring and ceiling of the sigmoid function. For a young normal‐hearing population, this is not necessarily beneficial because the range of SRT_beh_ is narrow, which is the case in the current study (see Figure 4B). However, if conducting a similar study with a population exhibiting a wider range of SRT_beh_, or, for example, evaluating a hearing‐aid algorithm, this approach could prove advantageous, because the informative range is measured. On the other hand, the approach presumes that the behavioral SRT is available, which cannot necessarily be assumed, especially when considering meaningful use cases of the EEG‐based SRT estimation. For instance, when measuring a non‐responsive population, the behavioral SRT cannot be measured, and it may be necessary to sample a wider range of SNRs than in the current study to find informative levels.

The range of reconstruction accuracies of the decoder (expressed as Pearson's correlations; see Figure 4A) in the current study is comparable to those reported in previous studies (Lesenfants et al. 2019; Vanthornhout et al. 2018). A statistically significant increase in reconstruction accuracies was observed when increasing the SNR in 2‐dB steps in the interval of SNR = SRT_beh_ − 4 dB to SNR = SRT_beh_ + 4 dB. This result indicates that the selected SNR range is relevant (with no flooring or ceiling effects) and that the 2‐dB step size is appropriate, as also supported by visual inspection of the reconstruction accuracy‐vs‐SNR data points in Figure 4A. However, the optimal step size may vary depending on the type of interfering signal and the characteristics of the population being tested, as both factors may influence the slope of the function.

It was not initially assumed that the SRT_neuro_ would be an unbiased estimate of SRT_beh_. However, no significant bias was observed, as the median of the individual difference between SRT_beh_ and SRT_neuro_ was very close to zero (0.38 dB). Consequently, no correction for a potential bias was applied. It is important to stress that this could be coincidental, and we do not claim that the SRT_neuro_ is entirely unbiased but that the two measures in this study had a very small difference. A bias correction could be necessary if changes in the acoustics or speech intelligibility measure were applied, for example, if a different speech test was used for the SRT_beh_, or if different stimuli were used for SRT_neuro_ determination.

### 4.2 TRF

The grand average TRF showed three distinct deflections within the time window from 0 to 400 ms, see Figure 5A. When observing the latency and amplitude for these deflections (see Figure 6), a general pattern emerged, with latencies decreasing and amplitudes increasing with increase in SNR. This pattern aligns with findings from a previous MEG study on normal hearing young adults (Ding and Simon 2013) and an EEG study on preschool children (Van Hirtum, Somers, Verschueren, et al. 2023).

When using mixed models to investigate the effect of SNR on the latency and amplitude of the individual deflections across all noise conditions (excluding the clean speech condition due to the undefined SNR), a significant decrease in the latency of N1 and P2 and a significant increase in the magnitude of the amplitude of the N1 and P2 were observed with increasing SNR (Table 3). However, interpreting changes in the individual peaks of the TRF should be done with caution because the amplitude and latency of the TRF peaks are often not uniquely tied to one underlying component. A change in the amplitude of a single underlying component may impact not only the amplitude and latency of the local TRF deflection but also those of the neighboring deflections. For instance, the P2 TRF peak can reflect changes in the N1 component. A delayed latency of the N1 component can contribute to a delayed latency and a smaller amplitude of the P2 peak. This is due to their opposite polarity and the fact that they overlap in time. Still, the increased latencies with a lowering of the SNR could suggest that more neural processing is needed when the SNR is low.

The SNR‐related changes in the TRF are interesting as they may be a result of the same changes in the underlying neural processes as the changes seen in the reconstruction accuracy. The decoder was trained on data obtained in the clean speech condition and as such fitted to predict the envelope in this condition. If the underlying neural processing changes, the decoder's estimate will, everything else equal, be worse. Therefore, if the neural processing changes when the SNR decreases, then that may likely be a part of the reason for the decrease in reconstruction accuracy. But even if the underlying neural processing was not changing, then the decrease in SNR would still result in a decrease in reconstruction accuracy, because the envelope that the brain entrains to is deteriorated by the noise.

Muncke et al. (2022) investigated the TRF responses at different SNRs in a normal‐hearing population using concatenated German matrix sentences from the Oldenburg Sentence Test (OLSA) as stimuli. The interferer consisted of random overlapping OLSA sentences. Their findings showed a negative deflection around 100 ms and a positive deflection around 200 ms, with an increasing amplitude and decreasing latency with increasing SNR, consistent with the results of the current study. Additionally, the N1 amplitude in the TRF has been shown to increase and its latency decrease with higher amplitude of the stimuli when using amplitude binning of the envelope (Drennan and Lalor 2019). This may also be part of the explanation for the increasing amplitudes and decreasing latencies observed with higher SNRs in this study, as modulation depth grows with increasing SNR. The previously observed increase in the decoders' reconstruction accuracy with higher SNR is likely connected to changes in the TRF, where greater SNR—and thus larger modulation depth—leads to higher amplitudes and shorter latencies. After all, whether using a forward or a backward approach, we are modeling the same underlying neural phenomena.

Notably, the prediction accuracy values in this study are higher centrofrontally compared to other scalp regions, and they increase with increasing SNRs (see Figure 5B). Similar observations have been reported in other EEG studies using encoding models with speech stimuli (Drennan and Lalor 2019; Fuglsang et al. 2017). We observed that the TRF had not converged to zero at 400 ms. For a validation check, we used an encoder with a longer time window (−100–1000 ms) and confirmed convergence (see Appendix S3).

### 4.3 Limitations

The reconstruction accuracy of the envelope is not a direct measure of speech intelligibility. However, previous studies have shown that the cortical entrainment to the envelope of speech is very robust to background noise, and the auditory cortex synchronizes to the speech rather than the physical presented mixture of speech and noise (Ding and Simon 2013). It has also been argued that the reconstruction accuracy of the envelope is correlated with speech intelligibility (Ding and Simon 2013; Iotzov and Parra 2019; Vanthornhout et al. 2018) and that the SRT_beh_ can be predicted from the reconstruction accuracy of the envelope (Lesenfants et al. 2019). A recent study (Karunathilake et al. 2023) suggests that the reconstruction accuracy of the envelope is not a direct speech intelligibility measure. Nonetheless, speech intelligibility and envelope reconstruction are still correlated, likely because the access to envelope information is essential for speech understanding (Shannon et al. 1995). Lesenfants et al. (2019) observed an improvement in SRT_neuro_ estimation when using an encoder with both phonetic and spectral features. Further research is needed to determine if these features could also improve predictions in the current dataset.

The current study investigated a population of young normal‐hearing participants, which results in a very narrow range of SRT_beh_ values and pure‐tone thresholds, see Figure 3B. Further research is needed to evaluate the methods used in the current study regarding individualized predictions in a more heterogeneous group exhibiting a wider range of SRT_beh_ values. Additionally, more research is needed to estimate the effect of different cognitive and auditory abilities on the TRF components and SRT_neuro_ estimation as the effect of cognitive and auditory abilities on the methods used in this study is unknown and previous studies have shown that cognitive and auditory abilities can influence speech comprehension (Dryden et al. 2017; Mukari et al. 2020; Nielsen and Dau 2011; Ren et al. 2019).

Some difference between the neural response of younger normal‐hearing and hearing‐impaired individuals must be expected. Age‐related changes in older normal‐hearing individuals include increased reconstruction accuracies, elevated TRF peak amplitudes, and different spatial patterns in prediction accuracy (Gillis et al. 2023; Karunathilake et al. 2023; Presacco et al. 2016). For the hearing‐impaired population, a delay in latency is expected, compared to age‐equivalent normal‐hearing individuals (Gillis, Decruy, et al. 2022). Investigating the overall feasibility of estimating SRT using continuous speech in young normal‐hearing individuals is a crucial first step. This approach avoids the potential additional variability induced by aging and hearing impairment, which could obscure the results and thus make it difficult to draw clear conclusions.

## 5 Conclusion

Pearson's correlation between the continuous speech envelope and the reconstructed speech envelope from EEG was used as a neural correlate of speech intelligibility. This was calculated at different SNR levels, which was manipulated by controlling the level of steady‐state speech‐shaped noise. The EEG‐based SRT (SRT_neuro_) was estimated by fitting a sigmoid function to the resulting reconstruction‐accuracy‐versus‐SNR data, which were compared against the behavioral SRT (SRT_beh_). The SRT_beh_ and SRT_neuro_ matched almost perfectly on the group level without an obvious bias between the two SRT measures. For 15 out of 20 participants (75%), the SRT_neuro_ was within 2 dB of the SRT_beh_, and for all participants (100%), it was within 3 dB. In addition, exploration of the TRFs showed a significant decrease in N1 and P2 latency along with a significant increase of magnitude in N1 and P2 amplitude with increasing stimulus SNR.

## Author Contributions

**Heidi B. Borges:** writing – original draft, data curation, formal analysis, funding acquisition, conceptualization, software, investigation, visualization, methodology. **Johannes Zaar:** writing – review and editing, funding acquisition, conceptualization, supervision, methodology, software, project administration. **Emina Alickovic:** writing – review and editing, funding acquisition, conceptualization, supervision, methodology, software. **Christian B. Christensen:** writing – review and editing, conceptualization, supervision, methodology. **Preben Kidmose:** conceptualization, methodology, writing – review and editing, supervision, project administration, funding acquisition.

## Conflicts of Interest

The authors declare no conflicts of interest.

### Peer Review

The peer review history for this article is available at https://www.webofscience.com/api/gateway/wos/peer‐review/10.1111/ejn.70083.

## Supporting information


**Figure S1** The temporal response function for each participant and condition. Data for different participants are depicted as thin lines in different colors; the mean data across participants are shown as bold black lines. The conditions are evident from the figure titles.
**Figure S2:** Example of the simulated data with the midpoint SNR of the simulated measurement grid equal to the behavioral SRT −3 dB, shown in red, and the actual reconstruction accuracies for the representative participant used for the simulation, shown in black.
**Figure S3:** Difference between the estimated sigmoid midpoint value from the simulated data and the behaviorally measured SRT for a representative subject (SRT_neuro_ − SRT_beh_) for each of the SNR sampling sets. The solid red lines show the medians, the edges of the blue boxes the 25th and 75th percentiles, and the black whiskers mark the most extreme values for non‐outliers. The red crosses mark datapoints considered outliers, defined as a value more than 1.5 times the interquartile range away from the bottom or top of the respective box. Differences from the individual simulations are shown as black circles and are jittered along the x‐axis to enhanced readability. The actually obtained SRT_neuro_ − SRT_beh_ for the representative subject is shown with a green cross.
**Figure S4:** The grand average TRF in a −100 to 1000 ms time window for each of the conditions.

## Data Availability

The data used in the study are available upon reasonable request to the corresponding author.
