# Supplementary material for: Speech Reception Threshold Estimation via EEG‐Based Continuous Speech Envelope Reconstruction
Source: Eur J Neurosci. 2025 Mar 27;61(6):e70083. doi: 10.1111/ejn.70083 (PMC11948451; doi:10.1111/ejn.70083)
Supplement: Supplementary file 1 — Figure S1 The temporal response function for each participant and condition. Data for different participants are depicted as thin lines in different colors; the mean data across participants are shown as bold black lines. The conditions are evident from the figure titles. Figure S2: Example of the simulated data with the midpoint SNR of the simulated measurement grid equal to the behavioral SRT −3 dB, shown in red, and the actual reconstruction accuracies for the representative participant used for the simulation, shown in black. Figure S3: Difference between the estimated sigmoid midpoint value from the simulated data and the behaviorally measured SRT for a representative subject (SRTneuro − SRTbeh) for each of the SNR sampling sets. The solid red lines show the medians, the edges of the blue boxes the 25th and 75th percentiles, and the black whiskers mark the most extreme values for non‐outliers. The red crosses mark datapoints considered outliers, defined as a value more than 1.5 times the interquartile range away from the bottom or top of the respective box. Differences from the individual simulations are shown as black circles and are jittered along the x‐axis to enhanced readability. The actually obtained SRTneuro − SRTbeh for the representative subject is shown with a green cross. Figure S4: The grand average TRF in a −100 to 1000 ms time window for each of the conditions. [file EJN-61-0-s001.pdf]

Appendix
S1: Temporal response functions for individual participants

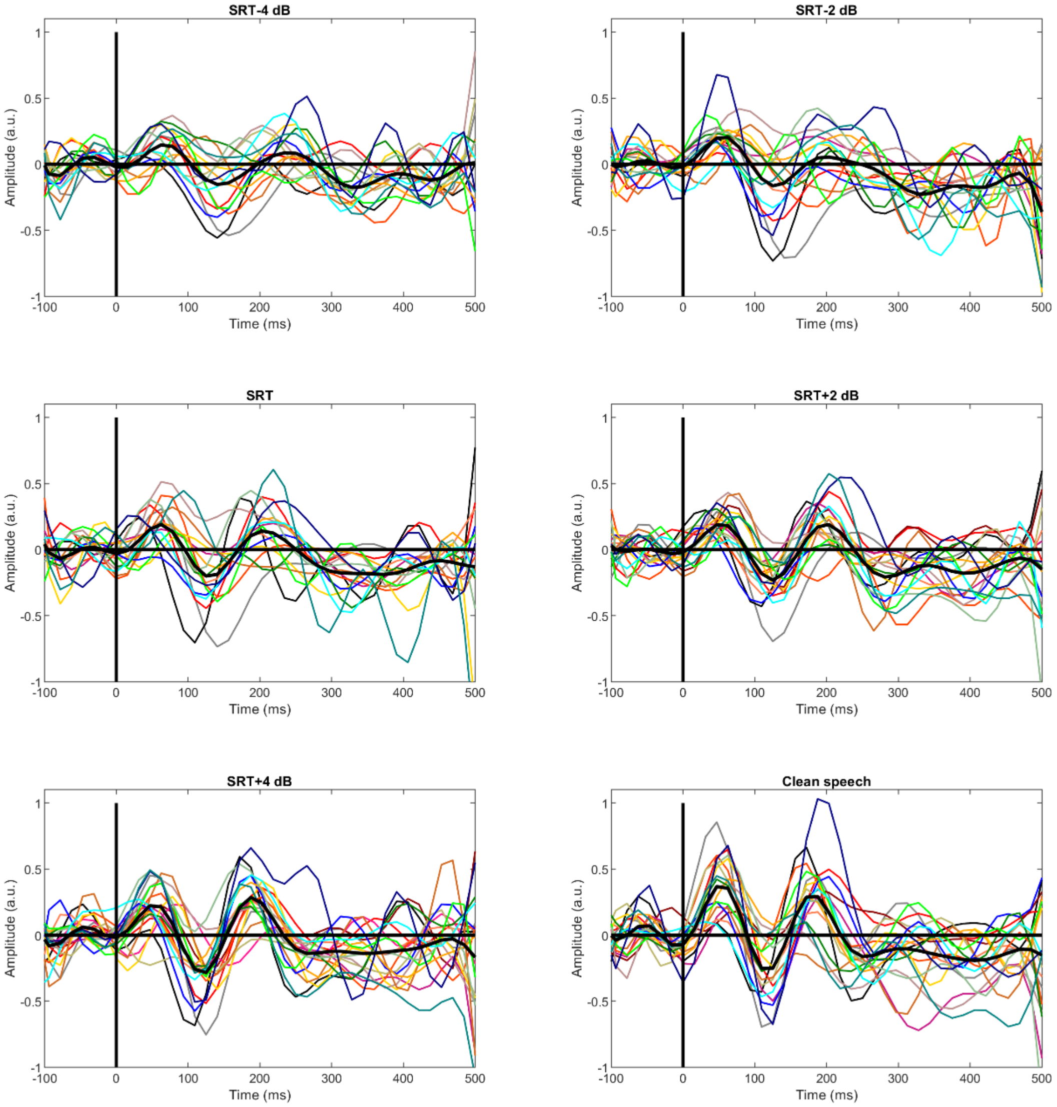

Figure S*1: The temporal response function for each participant and condition. Data for different participants are depicted as thin lines in different colors; the mean data across participants are shown as bold black lines. The conditions are evident from the figure titles.*

S2: Simulation of data with different SNRs

To investigate the potential systematic impact of the choice of SNR levels on the SRT estimation, a simulation was conducted. A new midpoint SNR was selected, ranging from -4 to +4 dB relative to the SRT_{beh} in 1-dB steps, resulting in 9 SNR sampling sets each consisting of five SNR values arranged in 2-dB steps around the respective midpoint SNR. For each of these 5 SNR levels, 16 reconstruction accuracy values were randomly sampled from a normal distribution. The standard deviation for this distribution was calculated using data from a representative participant, averaging the standard deviations across the 5 noise conditions. The mean of the normal distribution was determined using a sigmoid function fitted to the representative participant's data, with the reconstruction accuracy corresponding to each simulated SNR. This simulation was repeated 100 times for each SNR set, using the same parameters for p and b as the representative participant for the sigmoid fits. An example of the simulated data can be seen in Fig. S2.

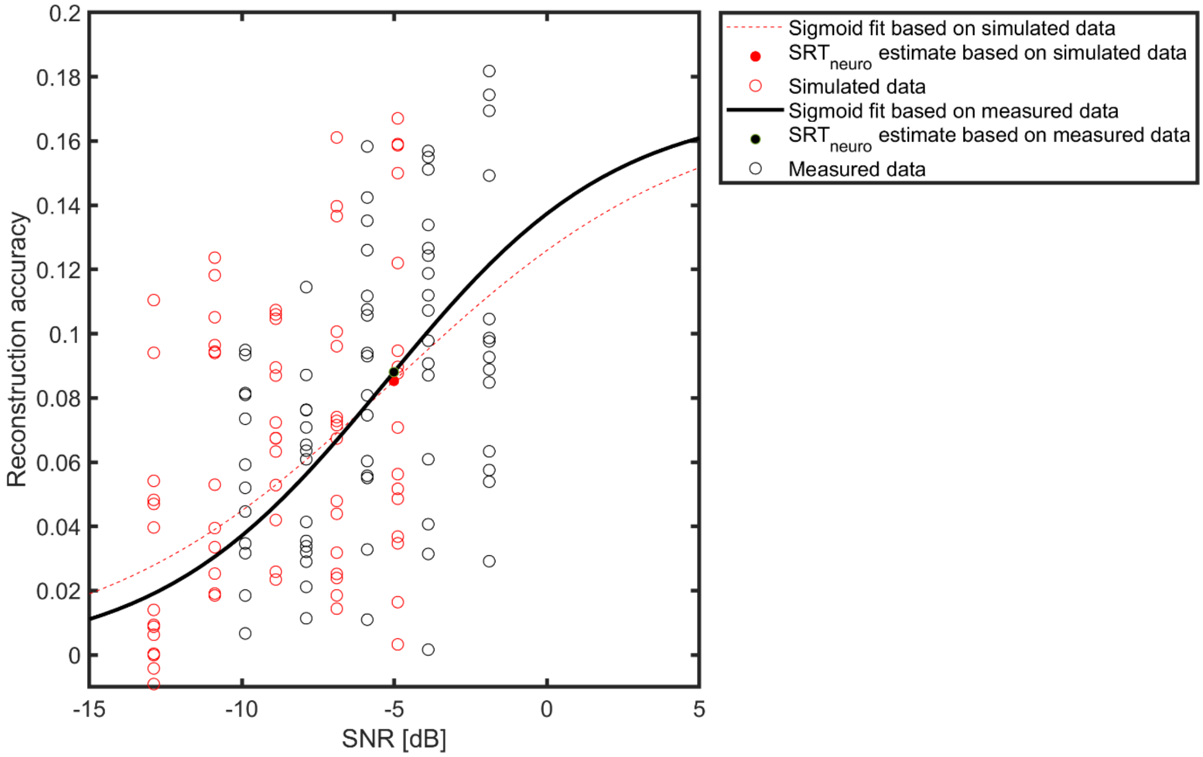

Figure S2: Example of the simulated data with the midpoint SNR of the simulated measurement grid equal to the behavioral SRT – 3 dB, shown in red, and the actual reconstruction accuracies for the representative participant used for the simulation, shown in black.

The difference between the SRT_{neuro} estimated from the simulated data and the measured SRT_{beh} for the representative subject is shown in Fig. S3. A one-way ANOVA was performed to test if the means of the differences between the simulated SRT_{neuro} and the measured SRT_{beh} were equal across the 9 SNR sampling sets. The p-value obtained was 0.20, indicating no statistical evidence that the SNR selection biased the sigmoid fit towards the measured SRT.

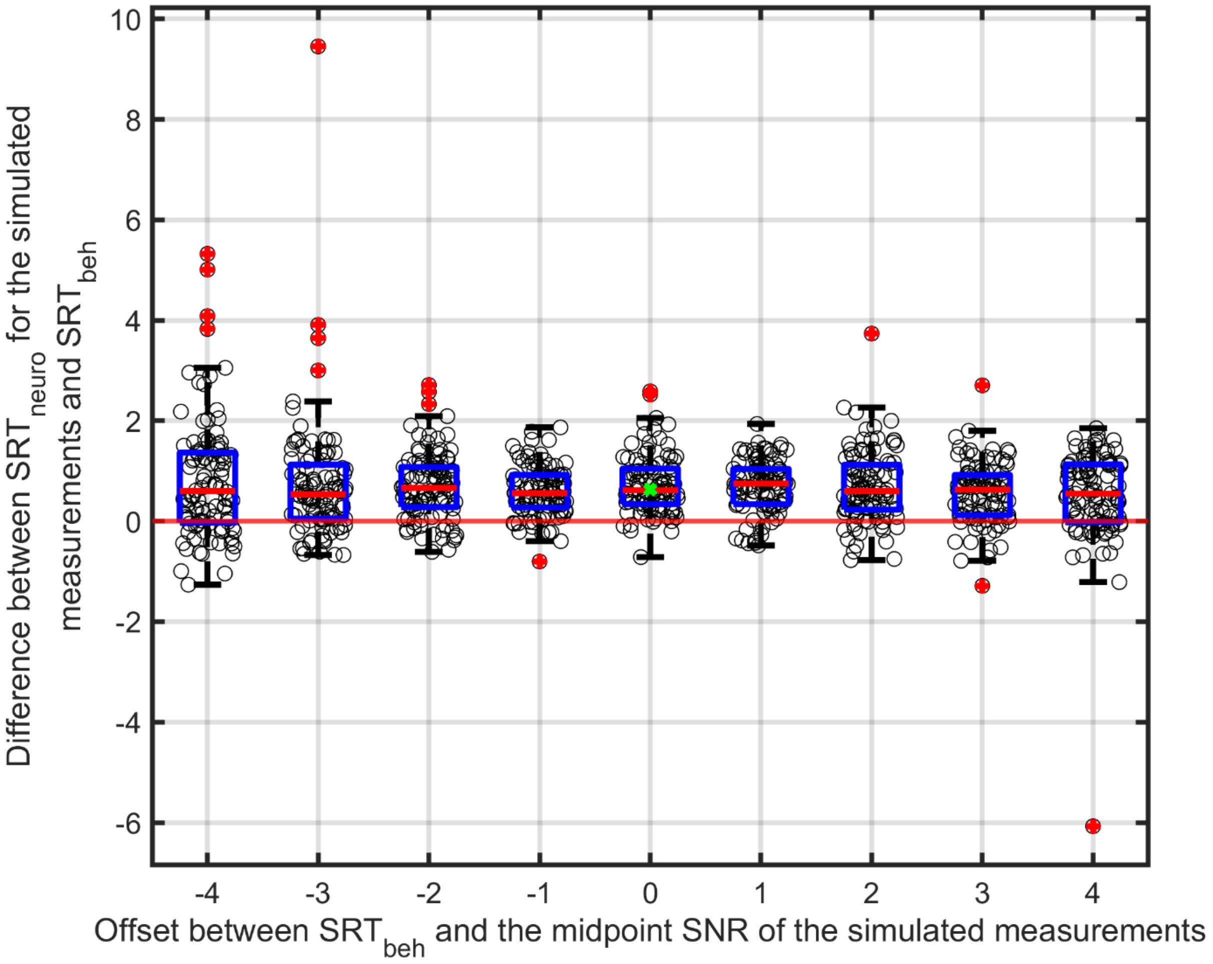

Figure S3: Difference between the estimated sigmoid midpoint value from the simulated data and the behaviorally measured SRT for a representative subject (SRT_{neuro} - SRT_{beh}) for each of the SNR sampling sets. The solid red lines show the medians, the edges of the blue boxes the 25th and 75th percentiles and the black whiskers mark the most extreme values for non-outliers. The red crosses mark datapoints considered outliers, defined as a value more than 1.5 times the interquartile range away from the bottom or top of the respective box. Differences from the individual simulations are shown as black circles and are jittered along the x-axis to enhanced readability. The actually obtained SRT_{neuro} - SRT_{beh} for the representative subject is shown with a green cross.

S3: Grand average temporal response functions with longer time window

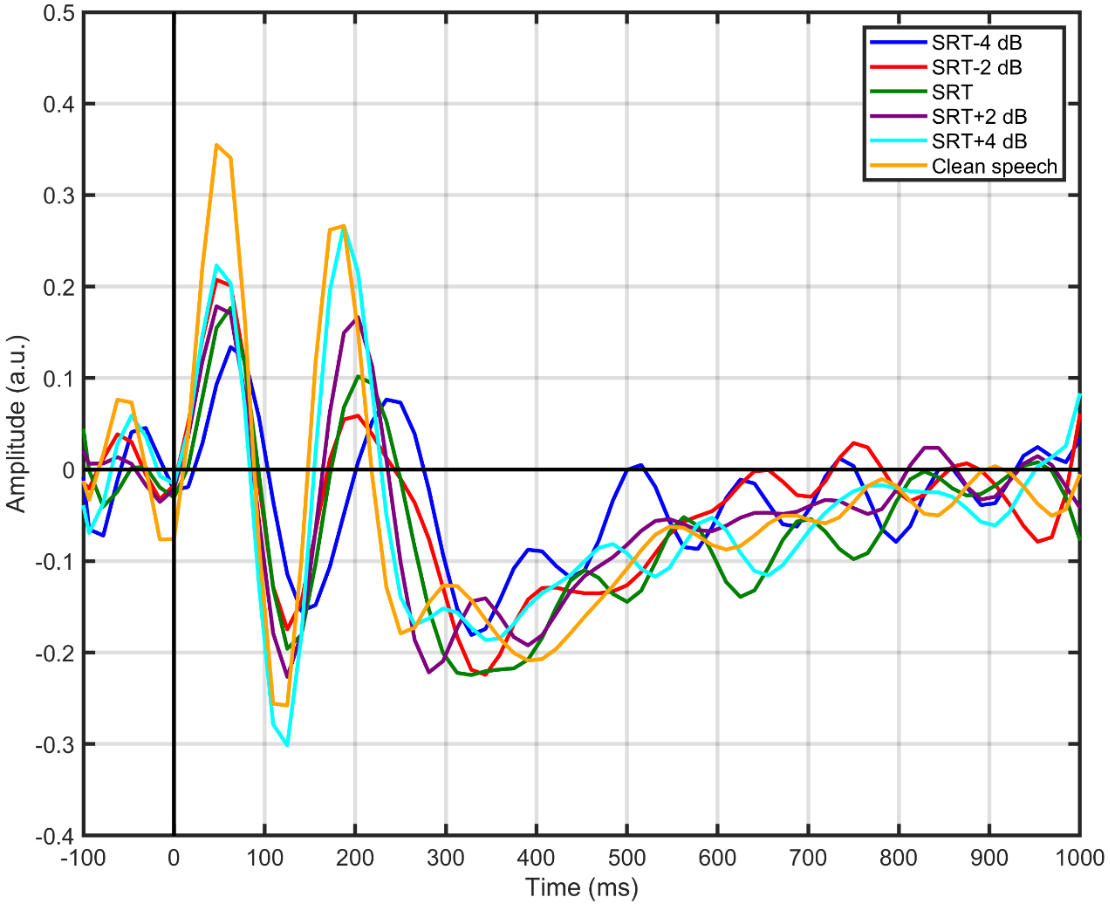

Figure S4: The grand average TRF in a -100 to 1000 ms time window for each of the conditions.
